# Involvement of school students in fights with weapons: prevalence and associated factors in Brazil

**DOI:** 10.1186/s12889-016-3629-1

**Published:** 2016-09-22

**Authors:** Alice Cristina Medeiros Melo, Leila Posenato Garcia

**Affiliations:** 1 University of Brasilia, SQN 203, Block C, apart 601. Asa Norte., ZIP: 70833-030 Brasilia, DF Brazil; 2 University of Brasilia and Institute of Applied Economic Research – Ipea, SBS 1, Block J., ZIP: 70076-900 Brasilia, DF Brazil

**Keywords:** Cross-sectional studies, Epidemiological surveys, School student health, Adolescent behaviour, Violence

## Abstract

**Background:**

Violence, as well as other behaviors, is often intensified during adolescence and early adulthood. The objective of this study is estimate the prevalence of Brazilian school students involvement in fights with weapons and to analyze the associated factors.

**Methods:**

This is a cross-sectional study using data from the National School Student Health Survey conducted in 2012 with 9^th^ grade elementary school students attending 2842 schools in all 27 Brazilian Federative Units. The outcome studied was involvement in fights with firearms and/or cold weapons in the 30 days prior to the interview. Poisson regression was used to estimate the prevalence ratios and 95 % confidence intervals (95 % CI). The analyses were stratified by sex.

**Results:**

Fifty seven thousand and eighty nine female students and 52,015 male students were included; the prevalence of their involvement in fights with weapons was 7.2 (95 % CI 6.9–7.5) and 13.8 (95 % CI 13.4–14.3), respectively. In the adjusted analysis the factors associated with male student involvement in fights with weapons were: being older, working, having smoked a cigarette, consumed alcoholic beverages and illicit drugs recently, insomnia, not having any close friends, skipping classes without parental supervision, having suffered aggression from a family member, reporting feeling unsafe on the way to or from school and/or at school. The same associated factors were found among female students in addition to not living with their father and/or mother and having suffered bullying. There was no association with type of school in either sex.

**Conclusion:**

Involvement in fights with weapons was greater among older male students. Health-risk behaviors, mental health characteristics, parental supervision and context of violence also showed association with the outcomes.

## Background

Internationally, adolescents are defined as young people between the ages of 10 and 19 years. The World Health Organization (WHO), in its report Preventing Youth Violence: an overview of the evidence, has defined youth violence as the one that occurs among people aged 10–29 [1].

Violence among adolescent and young adults is a global phenomenon [2]. Some 200,000 homicides of people aged 10–29 years occur every year, corresponding to the fourth leading cause of death in this age group worldwide [1].

Brazil is the world’s fifth largest country. In 2012 it had a population of approximately 194 million inhabitants, with 17.4 million (9.0 %) aged 10–14 and 52.2 (26.9 %) aged 15–29. Between 2000 and 2012, accidents and violence were the leading causes of death in this age group [3].

Weapons are frequently involved in severe and fatal injuries. In the United States of America (USA), around 18 % of adolescents in public and private schools in 2013 reported they had already carried weapons and 5.2 % reported carrying a weapon at school in the previous 30 days [4]. A study conducted in Argentina between 1991 and 2006 found that 48.5 % of fatal victims of firearm injuries were in the 15–29 age group [5].

A study carried out in public emergency services in Brazil in 2011 indicated that around one fifth of violence victims aged 15–29 had been injured by firearms [6]. A survey conducted in schools in 2009 revealed that 6.1 % of students reported a recent history of involvement in fights with cold weapons and 4 % in fights with firearms [7].

Previous studies that examined the factors associated with youth violence have shown the role of mental health characteristics [8, 9], alcohol consumption [10, 11], illicit drug use [12, 13], and family violence [14, 15]. Youth violence is a phenomenon with multiple causes and is associated to other forms of violence, including child maltreatment, intimate partner violence and self-harm [1]. Adolescents and young adults frequently are both victims and perpetrators of violence.

The objective of this study is to estimate the prevalence of Brazilian school student involvement in fights with weapons and to analyze the associated factors.

## Methods

This is a cross-sectional study using data from the second edition of the National School Student Health Survey conducted in Brazil in 2012. The survey was coordinated by the Brazilian Institute of Geography (IBGE) and the Ministry of Health.

The study population was comprised of 9^th^ grade public and private school students in state capitals and inner-state cities in all 27 Brazilian Federative Units. State capitals and Federal District formed 27 geographical strata, and the remaining cities were clustered within each of Brazil’s five main geographical regions, thus forming further five strata. A two-stage cluster sample was selected for the capital city and Federal District strata, whereby the first stage was comprised of the schools and the second stage was comprised of the eligible classes at the selected schools. In the strata formed by non-capital cities, the primary sampling units were the clusters of cities, the secondary sampling units were the schools and the tertiary sampling units were the classes at the schools. In both cases sampling was random and all students from the selected classes attending school on the day of data collection were invited to participate [16].

The sample was designed so as to enable population parameter estimates to be obtained for the capitals and the Federal District, the country’s five geographic regions (North, Northeast, Southeast, South and Midwest) and for Brazil as a whole. Sample size was calculated in order to provide estimated proportions for characteristics of interest in each of the geographic strata, with maximum error of 3 p.p. and a 95 % confidence level [16].

According to information obtained from the surveyed schools, a total of 132,123 9th grade students from the sampled classrooms were regularly attending classes, from which 110,873 were present on the date of the interviews (84 %). Additional losses included 1651 students who did not wish to participate and 118 who did not state their sex or age and were therefore excluded from the database. The final sample was comprised of 109,104 school students, from 2234 public (78.6 %) and 608 private (21,4 %) schools [16].

The survey questionnaire was self-administered. All students in each selected class were invited to respond using a smartphone. Most of the questions are closed and cover information on sociodemographic characteristics, eating habits, physical activity, smoking, alcohol and other drug use, body image and oral health, sexual behavior, violence and accidents.

For the purposes of analysis, this study outcome was defined by the combination of answers to the following questions: “In the previous 30 days, have you been involved in a fight in which someone used a firearm, such as a revolver or rifle? (no/yes)” and “In the previous 30 days, have you been involved in a fight in which someone used another form of weapon such as a knife, penknife, utility knife, stone, piece of wood or a bottle? (no/yes)”.

The outcomes were initially analyzed separately in order to find out whether there were differences between involvement in fights with firearms and with cold weapons, by sex. However, no substantial differences were found in prevalence nor in the associations. Therefore, we decided to analyze the combined outcome.

The following independent variables were included:

1. Sociodemographic characteristics:
  Age group (≤14 years; 15 years or over)
  Ethnicity/skin color (according to the official Brazilian classification: white; black; asian; brown and indigenous)
  Type of school (private; public)
  Employment – having a paid job (no/yes)
2. Health-risk behaviors:
  Cigarette smoking in the previous 30 days (no/yes)
  Alcohol intake in the previous 30 days (no/yes)
  Use of illicit drugs – such as cannabis, cocaine, crack, glue, ethyl chloride, ecstasy, oxy, etc. – in the previous 30 days (no/yes)
3. Mental health:
  Having insomnia – being unable to sleep at night because was very worried about something in the previous 12 months (no: never, rarely, sometimes; yes: often, always)
  Having close friends (one or more; none)
4. Parental supervision:
  Living with mother and/or father (no/yes)
  Skipping classes without permission in the previous 30 days (no/yes)
5. Context of violence:
  Suffering family violence – at least one episode of physical aggression perpetrated by an adult family member (no/yes)
  Suffering bullying – how often was insulted, mocked, scorned, intimidated or teased by school colleagues so much that felt hurt, upset, annoyed, offended or humiliated, in the previous 30 days (no: never, rarely, sometimes; yes: often, always)
  Feeling unsafe on the way to and from and/or at school – did not go to school because didn’t feel safe at least 1 day in the previous 30 days (no/yes)

The prevalence of the involvement in fights with weapons (firearms and/or cold weapons) and respective 95 % confidence intervals (95 % CI) were calculated, by sex, for all the categories of all the variables studied. The crude and adjusted prevalence ratios (PR) and respective 95 % CI were estimated using Poisson regression. All the independent variables were initially included in the adjusted analysis. Backwards selection was used. The final model comprised variables with Wald test p-values <0.05.

Data analysis was performed with Stata version 12.0 (StataCorp), using estimated proportional weightings to correct the probabilistic differences in the selection of students in each stratum and the effect of the sample design [16].

All the participants took part voluntarily after having given their free and informed consent, based on the autonomy of adolescents guaranteed by the Brazilian Statute of the Child and Adolescent (Law No. 8069/1990). The National School Student Health Survey (2012) survey was approved by the National Research Ethics Committee process number 16,805.

## Results

Of the 109,104 9^th^ grade students, 82.8 % attended public schools, 52.2 % were female and 68.3 % were aged 14 or under (Table 1).

**Table 1:** Characteristics of the 9^th^ grade students attending public and private schools in Brazil, 2012

	Total	
Variables	%	95 % CI
Sociodemographic characteristics		
Sex		
Female	52.16	(51.69–52.62)
Male	47.84	(47.38–48.31)
Age group		
< 11	0.00	(0.00–0.10)
12	0.73	(0.66–0.81)
13	22.13	(21.73–22.54)
14	45.55	(45.08–46.02)
15	18.35	(18.00–18.71)
16	8.37	(8.13–8.63)
17	3.19	(3.03–3.35)
18	0.97	(0.89–1.15)
≥ 19	0.67	(0.61–0.74)
Ethnicity/skin color		
White	36.78	(36.32–37.23)
Black	13.36	(13.03–13.69)
Yellow	4.10	(3.92–4.28)
Brown	42.24	(41.78–42.71)
Indigenous	3.53	(3.36–3.70)
Type of school		
Private	17.2	(16.8–17.5)
Public	82.8	(82.5–83.2)
Employment		
No	86.86	(86.54–87.16)
Yes	13.14	(12.84–13.46)
Health–risk behaviors		
Cigarette smoking in the previous 30 days		
No	94.93	(94.71–95.14)
Yes	5.07	(4,86–5.29)
Alcohol intake in the previous 30 days		
No	73.91	(73.49–74.32)
Yes	26.09	(25.68–26.51)
Use of illicit drugs		
No	97.60	(97.44–97.74)
Yes	2.40	(2.26–2.56)
Mental Health		
Insomnia		
No	90.30	(90.01–90.58)
Yes	9.70	(9.42–9.99)
Close friends		
One or more	96.50	(96.32–96.67)
None	3.50	(3.33–3.68)
Parental supervision		
Living with mother and/or father		
Yes	94.60	(94.40–94.80)
No	5.40	(5.20–5.60)
Skipping classes without permission		
No	74.19	(73.76–74.61)
Yes	25.81	(25.39–26.24)
Context of violence		
Suffering family violence		
No	89.40	(89.10–89.69)
Yes	10.60	(10.31–10.90)
Suffering bullying		
No	92.83	(92.58–93.08)
Yes	7.17	(6.92–7.42)
Unsafe on the way to and from and/or at school		
No	87.86	(87.5–88.2)
Yes	12.14	(11.8–12.5)

*95 % CI* 95 % confidence interval

**Fig. 1:**
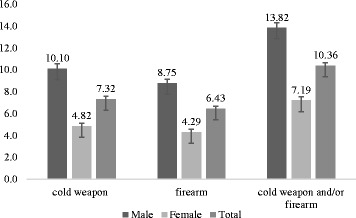
Prevalence (%) of history of involvement in fights with firearms and cold weapons among 9^th^ grade students attending public and private schools in Brazil, 2012

Prevalence of involvement in fights with weapons in the previous 30 days was 10.4 % (95 % CI 10.1–10.6). It was higher among male students (13.8 %; 95 % CI 13.4–14.3) than females (7.2 %; 95 % CI 6.9–7.5). Among males, prevalence of involvement in fights with cold weapons (10.6 %; 95 % CI 9.7–10.5) was higher than with firearms (8.8 %; 95 % CI 8.4–9.2), whereas among females there was no significant difference (Fig. 1).

Prevalence of involvement in fights with weapons was higher among older students. Among students aged up to 14 years old, the prevalence was 8.5 % (95 % CI 8.2–8.9), whilst among those aged 15 years or over it was 14.3 % (95 % CI 13.7–14.9). Especially among students aged 15 years or over, prevalence was higher among male than female students. The prevalence was higher in both sexes among students with black skin color and indigenous when compared to other ethnicity/skin color categories among which there was no significant difference (Table 2).

**Table 2:** Prevalence (%) of history of involvement in fights with firearm and/or cold weapon among 9^th^ grade students attending public and private schools in Brazil, 2012

	Male		Female		Total	
Variables	%	95 % CI	%	95 % CI	%	95 % CI
Sociodemographic characteristics						
Age group						
≥ 14 years	11.47	(10.91–12.04)	6.20	(5.84–6.58)	8.54	(8.22–8.87)
15 or over	17.96	(17.12–18.83)	9.80	(9.14–10.57)	14.30	(13.74–14.88)
Ethnicity/skin color						
White	13.20	(12.45–13.98)	6.39	(5.88–6.94)	9.81	(9.35–10.29)
Black	16.60	(15.30–17.98)	8.53	(7.47–9.72)	13.01	(12.13–13.94)
Yellow	14.75	(12.34–17.54)	7.40	(6.00–9.08)	10.62	(9.27–12.15)
Brown	12.95	(12.25–13.69)	7.12	(6.65–7.63)	9.66	(9.25–10.09)
Indigenous	16.78	(14.30–19.59)	11.11	(9.01–13.62)	13.83	(12.18–15.67)
Type of school						
Private	12.01	(11.01–13.08)	5.31	(4.64–6.07)	8.61	(8.00–9.26)
Public	14.21	(13.68–14.75)	7.56	(7.19–7.95)	10.72	(10.40–11.05)
Employment						
No	11.94	(11.45–12.45)	6.60	(6.27–6.95)	9.03	(8.74–9.33)
Yes	22.78	(21.45–24.17)	12.94	(11.63–14.37)	19.18	(18.19–20.20)
Health–risk behaviors						
Cigarette smoking in the previous 30 days						
No	12.12	(11.67–12.59)	6.25	(5.93–6.57)	9.05	(8.78–9.33)
Yes	45.34	(42.31–48.40)	25.00	(22.47–27.71)	34.83	(32.80–36.93)
Alcohol intake in the previous 30 days						
No	9.18	(8.73–9.65)	4.92	(4.59–5.26)	6.98	(6.70–7.27)
Yes	27.51	(26.30–28.77)	13.38	(12.56–14.24)	19.91	(19.18–20.66)
Use of illicit drugs						
No	12.5	(12.01–12.94)	6.64	(6.32–6.70)	9.41	(9.10–9.69)
Yes	59.1	(55.13–63.00)	33.70	(29.30–38.41)	48.60	(44.93–51.19)
Mental Health						
Insomnia						
No	13.07	(12.59–13.56)	6.30	(5.97–6.64)	9.65	(9.36–9.95)
Yes	24.38	(22.10–26.82)	13.24	(12.05–14.53)	16.71	(15.60–17.87)
Close friends						
One or more	13.29	(12.82–13.78)	7.05	(6.72–7.39)	10.00	(9.71–10.29)
None	24.03	(21.27–27.03)	11.97	(9.51–14.95)	19.53	(17.53–21.70)
Parental supervision						
Living with father and/or mother						
Yes	13.58	(13.11–14.08)	6.91	(6.58–7.26)	10.13	(9.84–10.43)
No	18.78	(16.57–21.21)	11.40	(9.88–13.11)	14.43	(13.13–15.83)
Skipping classes without permission						
No	10.99	(10.51–11.50)	5.46	(5.14–5.80)	8.03	(7.74–8.32)
Yes	21.09	(20.00–22.21)	12.67	(11.78–13.59)	17.02	(16.31–17.76)
Context of violence						
Suffering family violence						
No	11.82	(11.37–12.29)	5.93	(5.62–6.27)	8.78	(8.50–9.06)
Yes	32.11	(29.98–34.33)	16.85	(15.45–18.35)	23.47	(22.23–24.77)
Suffering bullying						
No	13.40	(12.92–13.9)	6.64	(6.32–6.98)	9.84	(9.55–10.14)
Yes	18.37	(16.59–20.29)	14.68	(12.85–16.72)	16.63	(15.34–18.01)
Unsafe on the way to and from and/or at school						
No	11.58	(11.13–12.06)	6.22	(5.90–6.56)	8.76	(8.48–9.05)
Yes	28.84	(27.04–30.69)	14.62	(13.30–16.05)	21.86	(20.71–23.05)

*95 % CI* 95 % confidence interval

Prevalence was also higher among public school students, those who worked, reported current smoking, alcohol use and illicit drug use, reported insomnia, had no close friends, did not live with their parents, skipped lessons without their parents’ knowledge, had suffered aggression by a family member, suffered bullying and those who had felt unsafe on the way to and from school or at school (Table 2).

In the adjusted analysis, we found an association between involvement in fights with weapons and being older, both among male (PR = 1.2; 95 % CI 1.1–1.3) and female students (PR = 1.2; 95 % CI 1.1–1.3). Students of both sexes who reported having paid jobs also had greater involvement in fights with weapons (males: PR = 1.3; 95 % CI 1.2–1.4; females: PR = 1.4; 95 % CI 1.2–1.5).

Among health-risk behaviors, it is noteworthy that prevalence of involvement in fights with weapons was approximately two times higher among males referring recent alcohol consumption (PR = 2.1; 95 % CI 1.9–2.2). Associations were also found with mental health variables, such as having no close friends (males: PR = 1.5; 95 % CI 1.3–1.6; females: PR = 1.4; 95 % CI 1.1–1.7), and not being able to sleep at night (males: PR = 1.2; 95 % CI 1.1–1.4 and females: PR = 1.4; 95 % CI 1.2–1.5). Regarding parental supervision, skipping classes without parents knowing was also associated with involvement in fights with weapons in both sexes (Table 3).

**Table 3:** Crude and Adjusted Prevalence Ratio (PR) of history of involvement in fights with firearm and/or cold weapon among 9^th^ grade students attending public and private schools in Brazil, 2012

	Sex							
	Male				Female			
Variables	crude PR 95 % CI	*p*	adjusted PR* 95 % CI	*p*	crude PR 95 % CI	*p*	adjusted PR* 95 % CI	*p*
Sociodemographic characteristics								
Age group		<0.001		<0.001		<0.001		<0.001
≥14 years	1		1		1		1	
15 or over	1.56 (1.46-1.68)		1.17 (1.09-1.25)		1.58 (1.44-1.74)		1.20 (1.09-1.32)	
Ethnicity/skin color		<0.001		0.056		<0.001		0.012
White			1		1		1	
Black	1.26 (1.14-1.39)		1.09 (0.99-1.20)		1.33 (1.14-1.56)		1.19(1.02-1.39)	
Yellow	1.12 (0.93-1.34)		0.90 (0.76-1.07)		1.16 (0.93-1.45)		1.11 (0.89-1.38)	
Brown	0.98 (0.91-1.06)		0.97 (0.89-1.04)		1.11 (1.00-1.24)		1.10 (0.99-1.22)	
Indigenous	1.27 (1.07-1.50)		1.10 (0.94-1.29)		1.74 (1.39-2.17)		1.44 (1.15-1.80)	
Type of school		<0.001				<0.001		
Private	1		-		1		-	
Public	1.18 (1.08-1.30)		-		1.42 (1.23-1.64)		-	
Employment		<0.001		<0.001		<0.001		<0.001
No	1		1		1		1	
Yes	1.91 (1.77-2.05)		1.32 (1.22-1.42)		1.96 (1.74-2.20)		1.35 (1.20-1.52)	
Health-risk behaviors								
Cigarette smoking in the previous 30 days		<0.001		<0.001		<0.001		<0.001
No	1		1		1		1	
Yes	3.74 (3.46-4.04)		1.41 (1.27-1.57)		4.00 (3.56-4.50)		1.58 (1.36-1.84)	
Alcohol intake in the previous 30 days		<0.001		<0.001		<0.001		<0.001
No	1		1		1		1	
Yes	3.00 (2.80-3.20)		2.07 (1.92-2.24)		2.72 (2.48-2.98)		1.77(1.60-1.97)	
Use of illicit drugs		<0.001		<0.001		<0.001		<0.001
No	1		1		1		1	
Yes	4.74 (4.39-5.12)		1.66(1.48-1.86)		5.08 (4.40-5.87)		1.70(1.43-2.03)	
Mental Health								
Insomnia		<0.001		<0.001		<0.001		<0.001
No	1		1		1		1	
Yes	1.87 (1.68-2.07)		1.23 (1.11-1.36)		2.10 (1.89-2.34)		1.37 (1.22-1.53)	
Close friends		<0.001		<0.001		<0.001		0.011
One or more	1		1		1		1	
None	1.81 (1.60-2.05)		1.45 (1.28-1.64)		1.70 (1.35-2.14)		1.36(1.07-1.72)	
Parental supervision								
Living with mother and/or father		<0.001						
Yes	1		-		1		1	
No	1.38 (1.22-1.57)		-		1.65 (1.42-1.92)		1.29 (1.11-1.50)	
Skipping classes without permission		<0.001		<0.001		<0.001		<0.001
No	1		1		1		1	
Yes	1.92(1.79-2.06)		1.30 (1.20-1.39)		2.32 (2.11-2.55)		1.46 (1.31-1.61)	
Context of violence								
Suffering family violence		<0.001		<0.001		<0.001		<0.001
No	1		1		1		1	
Yes	2.72 (2.51-2.94)		1.62 (1.49-1.76)		2.84 (2.57-3.14)		1.72 (1.54-1.93)	
Suffering bullying		<0.001				<0.001		<0.001
No	1		-		1		1	
Yes	1.37 (1.23-1.53)		-		2.21 (1.92-2.54)		1.55 (1.34-1.79)	
Unsafe on the way to and from and/or at school		<0.001		<0.001		<0.001		<0.001
No	1		1		1		1	
Yes	2.49 (2.31-2.68)		1.50 (1.38-1.63)		2.35 (2.11-2.62)		1.36 (1.21-1.54)	

*crude PR* crude prevalence ratio; adjusted, *PR* adjusted prevalence ratio, *95 % CI* 95 % confidence interval

There was a clear association between involvement in fights with weapons and context of violence. Students who referred having suffered aggression by a family member had higher prevalence of involvement in fights with weapons than those who did not report this type of aggression, both among males (PR = 1.6, 95 % CI 1.5–1.8) and females (PR = 1.7; 95 % CI1.5–1.9). Students of both sexes who reported feeling unsafe on the way to or from school and/or at school also had greater involvement in fights with weapons (Table 3).

In order to better explain the adjusted model, we have rewritten the paragraph, “In the crude analysis attending a public school, was shown to be a risk factor for involvement in fights with weapons in both sexes. After adjustment, this variable did not meet the criteria to be part of the final model. Not living with the father and/or mother and having suffered bullying also showed association with the outcome in the crude analysis. However, these variables didn’t enter in the final model for male students (Table 3).

## Discussion

Prevalence of involvement in fights with firearms and cold weapons was considered to be high, given that approximately 1 in 10 school students reported at least one of these outcomes in the 30 days prior to the interview. Older male students showed higher prevalence, as did those who reported paid work. Health-risk behaviours, mental health characteristics, parental supervision and situations of violence were also associated with the outcomes.

Consistently with studies that reported a higher prevalence of carrying weapons among males, we found that the prevalence of male students involvement in fights with weapons was almost twice that of females. A study conducted in 2006 in Belgium, Israel, the USA, Canada and Macedonia with students aged 11 to 15 found that having carried weapons in the 30 days prior to the survey varied between 11.3 % (Belgium) and 22.2 % (USA) among boys and 1.6 % (Belgium) and 7.1 % (USA) among girls, showing the disparity between the sexes [8]. A survey with school students carried out in 2013 in the USA showed that 17.9 % had carried weapons in the 30 days prior to the survey and prevalence was around four times higher among male students [4]. Differences between the sexes in relation to patterns of morbidity and mortality as a consequence of models of masculinity have been widely discussed in the literature [17].

We also found that older students had greater participation in fights with weapons. Similarly, a study undertaken in 32 countries of the Americas between 1999 and 2009 showed a risk of death from homicide almost 20 times greater (RR = 19.3; 95 % CI 18.9–19.8) among young people aged 15–24 when compared to those aged under 15 [18]. On the other hand, a study conducted with school students from 43 countries in Europe and North America in 2009 and 2010, found that prevalence of physical fighting three or more times during the 12 months prior to the study was lower among older students (14 % at age 11 and 10 % at age 15) [19].

It is noteworthy when considering the specificities of older adolescents that Brazil’s Youth Statute came into force in 2013. This defines youth (15-29 years) as the target population for public policies that encompass the promotion of a safe life, and a culture of peace, solidarity and non-discrimination [20].

Adolescents who worked had greater prevalence of involvement in fights weapons. Starting work may lead to adolescents taking on adult roles early, in addition to heightening health-risk behaviours [21]. A study with Brazilian school students found that those who were working were more likely to consume alcohol, drive a motor vehicle and to have been involved in fights [22]. However, we should consider the fact of students getting involved in fights may be a reason for their parents encouraging them to get a job.

Students who reported use of cigarettes, alcohol and illicit drugs had higher prevalence of involvement in fights with weapons. Resnick *et al*. [23], in a longitudinal study conducted in USA, found that frequent use of alcohol, cannabis and other illicit drugs was strongly associated with violence among students.

The 2013, the global burden of disease study showed that alcohol consumption (10 %) is the main risk factor for disability adjusted life years (DALYs) among people aged 10–24. Among males, alcohol and illicit drug use together accounted for around 23 % of the global burden of disease in the South American region [24]. Harmful use of alcohol and illicit drugs appears as a risk factor at both individual and community level, given that the latter provides the local supply of weapons and illicit drugs, as well as facilitating access to alcohol [1].

We must point out that in Brazil the sale of alcoholic beverages to people aged under 18 is prohibited, although frequently the interests of the industry override those of public health. Little rigour is applied to the enforcement of legal measures and this in turn results in alcohol consumption beginning at an early age among school students in Brazil [22]. Several studies confirm the severe problem of alcohol consumption among adolescents in Brazil [25–29].

We also found an association between involvement in fights with weapons and mental health characteristics. Similarly, a study conducted in 2006 with school students aged 11–15 in five countries found positive and statistically significant associations between carrying weapons and emotional health characteristics, such as irritability and difficulty in sleeping [8]. A study conducted with students (aged 12–18 years) in Vietnam found association between multiple types of maltreatment and mental health (depression, anxiety and low self-esteem) [30].

Our study findings agree with those of other studies undertaken in Brazil and in other countries with regard to parental supervision being a protective factor against health problems among school students [30–33]. A study conducted in 2009 with Brazilian school students, found a dose–response gradient: if a student skipped school without permission for 1 or 2 days, the likelihood of experimenting drugs increased 1.9 times; whilst among those who skipped school 3 days or more, the likelihood increased 4.3 times [34].

The association between involvement in fights with weapons and not living with parents and suffering bullying was found in the adjusted analysis only for females. These findings may be related to the fact that girls tend to get involved in fights as a defence against provocation or aggression and have less support from their family [35, 36]. Studies show that girls are the main victims of bullying, or at least tend to report more victimization than boys [37, 38].

In the crude analysis, attending a public school was shown to be a risk factor for involvement in fights with weapons in both sexes. In Brazil, studying in a private or in a public school is a proxy of socioeconomic status, with students from lower income families studying in public schools, and those from higher income families studying in private schools. However, this variable did not enter the final model, suggesting that the social and family context surrounding adolescents have a major role. Also, in the Brazilian social context it is possible that poorer public school students could seek employment as a way of living, and also to become separated from their families and close friends.

Moreover, the prevalence was higher among students who suffered aggression from a family member or reported feeling unsafe at school or on the way. A study comparing the results of the 2009 and 2012 National School Student Health Surveys in Brazil found a significant increase in the proportion of students experiencing situations of violence in the two places that should ensure their healthy and safe development: school and home [15]. According to the World Health Organization, interpersonal relationships – such as family, friends and colleagues – can also strongly affect violent behaviour in adolescents [2], building an vicious circle of violence. It has also been shown that experiences of physical violence, and other factors included in this study, such as bullying and loneliness, limited parental support and alcohol and tobacco use, were associated with suicidal ideation [39].

We must point out that this study has limitations related to the survey design. It included only adolescents attending school and who were in the classroom on the day the questionnaire was administered, what may have caused selection bias, given that school absenteeism may be related to the outcomes and factors studied. Nevertheless, it is appropriate to emphasize that Elementary Education is universal in Brazil. The estimated school attendance rates for 6–14 year olds in 2012 was 98.2 % [40]. As this was a cross-sectional study, it is not possible to determine the temporal sequence between the events. For instance, it is not possible to assess whether consuming illicit drugs or having suffered physical aggression from a family member is a cause or a consequence of adolescent involvement in fights with weapons.

No assessment has been made of the influence of the self-reported data collection method with the use of smartphones, specifically with regard to involvement in fights with weapons, use of drugs, etc., on the validity of the information collected. However, given that the data was collected without an interviewer and anonymously, we believe that prevalence underestimation is minimum. On the other hand, as fights are events that stand out, some students may have replied positively about their occurrence even when they happened prior to the 30-day period specified in the question.

## Conclusions

Notwithstanding its limitations, this study provided input regarding the prevalence of school students’ involvement in fights with weapons and its associated factors in Brazil. Our results reinforce the importance of the Health at School Programme [41] and other violence prevention and health promotion actions for Brazilian youth including the promotion of a culture of peace. Our findings support that prevention strategies should focus on students who present school failure, work, and exhibit health-risk behaviours – smoking, alcohol drinking, and use of illicit drugs. Family support and supervision are also important for the prevention of student violence.

## Abbreviations

95 % CI: 95 % confidence intervals
DALYs: Disability adjusted life years
IBGE: Brazilian Institute of Geography
PR: Prevalence ratio
USA: United States of America
